# Thymidylate Synthase Expression Determines Pemetrexed Targets and Resistance Development in Tumour Cells

**DOI:** 10.1371/journal.pone.0063338

**Published:** 2013-05-13

**Authors:** Aitziber Buqué, Unai Aresti, Begoña Calvo, Jangi Sh. Muhialdin, Alberto Muñoz, Sergio Carrera, Eider Azkona, Itziar Rubio, Guillermo López-Vivanco

**Affiliations:** 1 Medical Oncology Research Laboratory, Cruces Universtity Hospital, Bizkaia, Spain; 2 Medical Oncology Department, Cruces Universtity Hospital, Bizkaia, Spain; Virginia Commonwealth University, United States of America

## Abstract

Although treatment options for cancer patients are increasing every year, the drug resistance problem remains very present. It is very difficult to find a drug that acts equally on tumours of the same histology as the individual's genetic characteristics often determine the response to treatment. Furthermore, tumours that initially respond to anti-tumour therapy are able to adapt and develop resistance to the drug, while others do not. In addition, this usually implies resistance development to agents to which the cells have not been exposed, a phenomenon called cross-resistance or multidrug resistance. Given this situation, it has been suggested that the most appropriate treatment would be able to act in parallel on multiple pathways constitutively altered in tumour cells. Pemetrexed is a multitargeted antifolate that exerts its activity against folate-dependent enzymes involved in *de novo* pyrimidine and purine synthesis. It is currently in use in combination with cisplatin against malignant pleural mesothelioma and non-squamous non-small cell lung cancer with favourable results. By real-time RT-PCR gene expression assays and restoration viability assays we demonstrated that Pemetrexed targets folate-dependent enzymes involved in *de novo* biosynthesis of purines differently depending on the intrinsic genetic characteristics of the tumour. These differences did not, however, interfere either with the initial response to the drug or with the activation of apoptotic pathways. In addition, these genetic fingerprints can differentiate two groups of tumours: those capable of developing resistance to antifolate, and not capable. These results may be useful to employ targets gene expression as resistance markers, a valuable tool for identifying patients likely to receive combination therapy to prevent the development of resistance.

## Introduction

There is increasing awareness of the importance of genetic background in individual susceptibility to cancer treatments. In human populations, multiple genetic parameters have been associated with response or resistance to chemotherapeutic agents: SNPs, gene copy number or gene expression. Interindividual variation in biological responses to anticancer drug could be a result of internal and external alterations. On one hand, the intrinsic characteristics of each tumour enable them to be sensitive or refractory to the agent employed. This is the case of inherent drug resistance to some antifolates where alterations of the reduced folate carrier (RFC) role results in the impaired drug uptake. [1].

In addition, cancer treatments are often administered cyclically to allow recovery of the patient. But this scheme also makes possible the tumour cell recovery and adaptation. The treatments do not always manage to eradicate all malignant cells, enabling tumour cells to adapt their genetic characteristics in order to achieve a survival advantage [2], [3]. These new genetic alterations are usually treatment-specific, and in certain instances are common to various tumour types. In most cases, modifications involve the overexpression of target molecules, downregulation of proapoptotic factors, upregulation of prosurvival mediators, or deregulation of genes involved in the DNA damage detection and repair systems [1]. This is the case for the regulation that p63 exerts on Akt, where Akt activity induces survival of cancer cells upon cisplatin exposure [3]. Active Akt is able to regulate several molecules involved in cell survival, both directly and indirectly, through mammalian target of rapamycin (mTOR) which is responsible for controlling the cell cycle, apoptosis and growth via the regulation of several downstream proteins [4]–[10].

Both inherent and acquired resistance has become a major challenge for the oncologist. That is why combined agents and drugs having multiple targets are being used aimed to simultaneously act on multiple constitutively modified pathways that confer a survival advantage to tumor cells, and on key factors weakening the malignant cells to prevent the new resistance development.

Pemetrexed (Alimta®, MTA) is a multitargeted antifolate which inhibits folate-dependent enzymes involved in *de novo* biosynthesis of purines and pyrimidines. MTA acts as a potent inhibitor of thymidylate synthase (TS), and this enzyme is defined as its primary target [1]. On the other hand, there is not a clear consensus on the secondary targets of MTA in different tumours. While dihydrofolate reductase (DHFR) is well recognised as a secondary target, the site of action of MTA on purine synthesis is a source of discussion. Some authors class both glycinamide ribonucleotide formyltransferase (GARFT) and aminoimidazole carboxamide ribonucleotide transformylase (AICART) as secondary targets, while studies with CCRF-CEM human lymphoblastic leukaemia cells indicate that AICART, but not GARFT, is a secondary target of MTA [11]. In particular, there was found to be an accumulation of the substrate of AICART, aminoimidazole carboxamide (AICA, also called ZMP), causing activation of adenosine monophosphate-activated protein kinase (AMPK) and hence inhibition of the mTOR pathway, which is responsible for balancing energy metabolism, protein and lipid synthesis, and cell growth [11].

The adequate definition of MTA targets is a relevant point to better understand clinical responses to the drug and explore potential mechanisms of resistance development.

## Materials and Methods

### Cells and Culture Condition

A375, Hs294T, HT144 and MeWo human melanoma cell lines and the Calu-3 NSCLC cell line were obtained from the American Type Culture Collection (ATCC, Rockville, Maryland). A375, HT144 and Hs294T were maintained in DMEM cell culture medium supplemented with 10% FBS (Cambrex Bio Science, Verviers, Belgium) as recommended by the ATCC. MeWo melanoma and Calu-3 lung adenocarcinoma cell lines were maintained in Eagle Minimum Essential Medium (EMEM, Cambrex Bio Science, Verviers, Belgium) with 2 mM L-glutamine (Cambrex Bio Science, Verviers, Belgium) and Earle's BSS adjusted to contain 1.5 g/L sodium bicarbonate, 0.1 mM sodium pyruvate and 10% FBS as described by the ATCC. Table 1 summarizes some of the genetic characteristics of the cell lines studied.

**Table 1:** Table summarizing the genetic characteristics of studied cell lines.

	A375	Hs294T	HT144	MeWo	Calu-3
**BRAF**	c.1799 T>A	wt	c.1799 T>A	wt	wt
**NRAS**	wt	wt	wt	wt	wt
**aDKN2a**	c.181G>Tc.205G>T	c.1 150del150	c.1 471del471	c.237 238CC>TT	c.247C>T
**cDKN2a(p14)**	c.1 316del316	wt	c.1 522del522	wt	wt
**PTEN**	wt	wt	c.165 209del45	wt	wt
**NF1**	wt	wt	wt	c.4006C>T	wt
**TP53**	wt	wt	wt	c.772G>Ac.949C>T	c.711G>T
**SMARCA4**	wt	wt	wt	wt	wt

According to COSMIC database from **Sanger Institute** and “**The TP53 web site**”.

MTA (Lilly France, Fegersheim, France) was dissolved in phosphate buffered saline (PBS) at 2 mg/ml and stored at –80°C until use.

### Determination of MTA Targets

**Figure 1:**
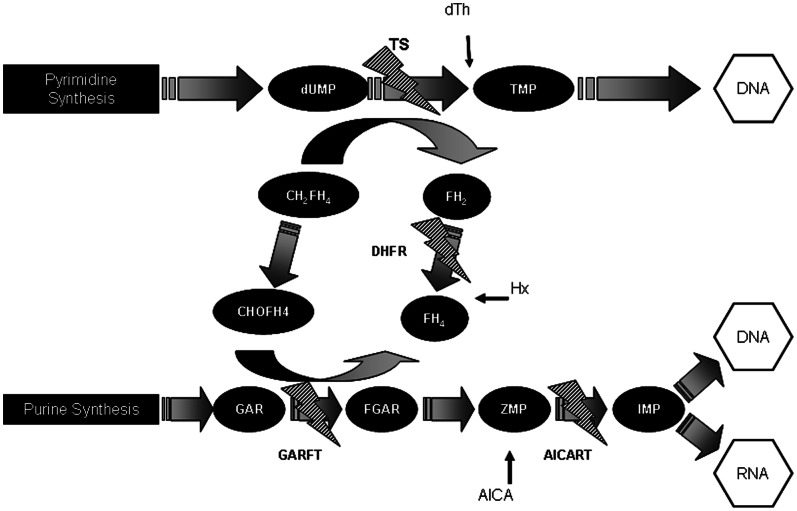
A schematic diagram of the pyrimidine and purine biosynthesis pathways is shown, where the MTA-targets are indicated with a lightning bolt symbol. The effect of the addition to the culture medium of the preformed purine Hx, the preformed pyrimidine dTh and/or the purine pathway intermediate AICA as a new source for the restoration of the MTA-interrupted biosynthesis of purines and pyrimidines was different depending on the cell line.

For each cell line, 10,000 cells were plated in 96-well plate and allowed to adhere for 24 h at 37°C and 5% CO_2_. As previously described [11], we added appropriate intermediary metabolites alone or in combination to each well, to restore purine and/or pyrimidine biosynthesis pathways (Figure 1). Thymidine (dTh) was used at a range of 12–200 µM, hypoxantine (Hx) at 50–800 µM and aminoimidazolecarboxamide (AICA) at 400–3,000 µM. Cell viability was determined by XTT.

### Gene Expression Assays

The mRNA was extracted from cell lines with the RNeasy Plus Mini Kit (Qiagen, cat. no. 74134). Gene expression levels were measured in each sample by one-step real-time RT-PCR using human validated TaqMan Gene Expression Assays: GARFT (Hs00531926_m1), AICART (Hs00269671_m1), TS (Hs00426586_m1), FPGS (Hs00191956_m1), DHFR (Hs00758822_s1) and RFC (Hs00161870_m1) and the human RPLPO was used as endogenous control, all of them from Applied Biosystems. Three replicates were run for each sample in a 96-well and 200 ng RNA were used for each reaction. Results were analysed using the ΔΔCt method.

### Flow Cytometry

In each case, 500,000 4% formalin-fixed cells were washed twice in 1×PBS and permeabilized with ice-cold 70% ethanol for 30 min. Cells were labelled with 25 µg/ml in 1×PBS of anti-PAkt at S473 (R&D Systems, cat. no. AF887) for 30 min on ice. Cells were washed with 1×PBS and incubated with secondary antibody anti-rabbit-Alexa 488 (Invitrogen, cat. no. A24922) for 30 min on ice and in the dark. Finally, cells were washed with 1×PBS and 5,000 cells were analyzed on a Cytomics FC 500 MPL cytometer (Beckman Coulter).

### SDS-PAGE and Immunoblot

MTA-treated and untreated human melanoma cells were harvested by trypsinization. Total cell lysate was isolated by resuspending pelleted cells on ice cold Igepal-Triton lysis buffer (1% Igepal, 1% Triton X-100, 20 mM Tris pH 7.3, 140 mM NaCl, 1 mM EDTA all from Sigma and completed with water) containing protease and phosphatase inhibitors (3% protease inhibitor cocktail, 10 mM NaF, 10 mM NaPPi, 1 mM Sodium Vanadate, 1 mM phenylmethylsulfonyl fluoride [PMSF], all from Sigma) followed by 30 minutes incubation on ice and vortexed every 10 min. Aliquots of lysate were resuspended in Laemmli sample buffer (Bio-Rad Laboratories), resolved by SDS-PAGE and transferred to PVDF membrane with the iBlot® Gel Transfer Device as described by the manufacturer (Invitrogen Life Technologies). The following antibodies were used in immunoblotting: anti-actin (1∶200) from Sigma, Ph-4E-BP1 at Thr37/46 (Cell Signalling, cat. no. 236 B4) mAB and horseradish peroxidase (HRP)-conjugated secondary antibodies (1∶5,000; Pierce Biotechnology).

### Resistance Induction

Tumour cells were plated at a density of 1.5×10^6^ cells. After 24 hours, the medium was changed, and 20% of their respective IC_50_ of MTA was added. Thereafter, the medium was changed every 2 days, and when cell reached 80% confluence the MTA concentration was doubled. This was repeated gradually increasing the MTA concentration (up to 1.7 µM in the last cycle). If cell confluence fell to less than 20%, the medium was changed to drug-free medium or medium with the previous dosage. Resultant cell lines were designated as A375-M, Hs294T-M and MeWo-M.

## Results

### Pathway Restoration

**Figure 2:**
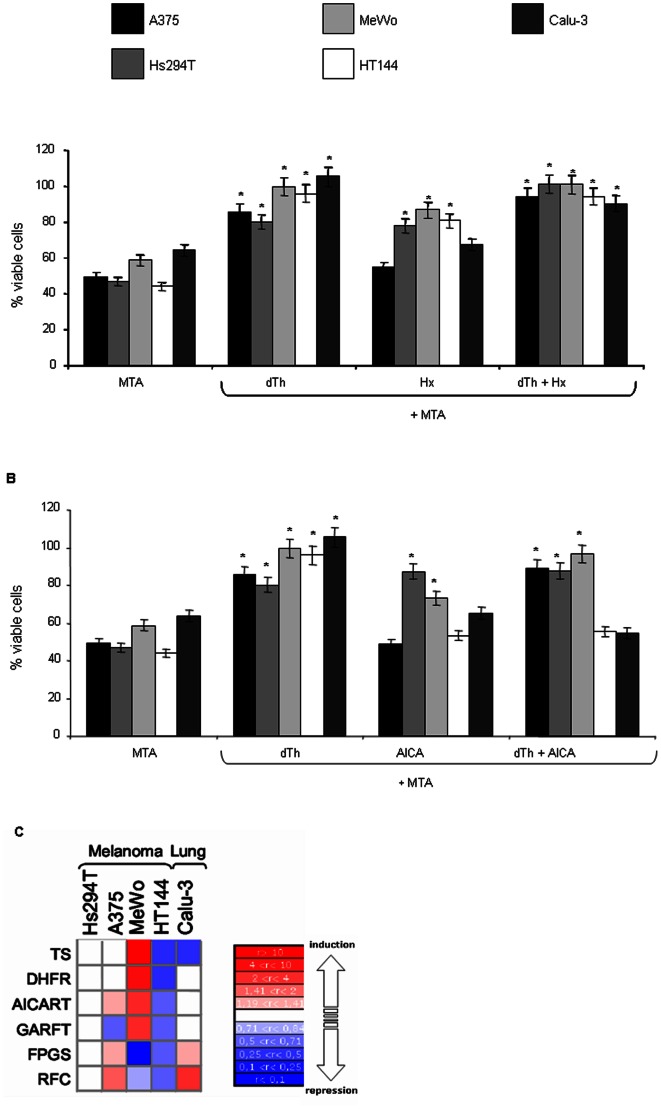
Viability and proliferation XTT after 48 h of exposure to MTA alone or in combination with dTh, Hx, or/and AICA. The percentage of viable cells is shown relative to viability of MTA-unexposed cells (control conditions). These results are representative of three independent experiments. **A)** Viability assays before and after MTA exposure with the pyrimidine biosynthesis pathway restored by addition of Hx alone or in combination with dTh. **B)** Viability assays before and after MTA exposure with purine biosynthesis pathway restored through the addition of AICA alone or in combination with dTh. **C)** Heatmap of six MTA-related genes where up- and down-regulation fold changes corresponding to each colour are indicated on the scale on the right of the figure.

XTT cell viability assays revealed that MTA-induced growth inhibition was not completely blocked by the preformed purine Hx in all cell lines (Figure 2A), but inclusion of dTh in the culture medium in order to eliminate the effect on the primary target TS, reversed the growth inhibitory effects.

The restoration of the purine biosynthesis was not, however, so homogeneous, responses to the assay varying between the cell lines studied (Figure 2B). Including AICA in the growth medium made available a purine pathway intermediate that is downstream of GARFT but upstream of AICART. Therefore, assuming GARFT is inhibited by MTA, the inclusion of AICA in the growth medium should reduce growth inhibition. In fact, this seemed to be the case for A375, Hs294T and MeWo cell lines, although the suppression of the cytotoxic activity was not complete, as with Hx. Further, the inclusion of dTh together with AICA fully suppressed MTA-induced cell death in these cell lines (A375, Hs294T and MeWo).

On the other hand, either no effect or a mild increase in growth inhibition was obtained for HT144 and Calu-3. In these cases, the failure to restore the pathway suggests that MTA disrupts the pathway by targeting an enzyme downstream of AICA, i.e. AICART, regardless of its effect on GARFT.

### Target Gene Expression

In order to assess a possible correlation between enzyme expression and the secondary target, the expression level of MTA-related enzymes as well as reduced folate carrier (RFC) and the folylpolyglutamate synthase enzyme (FPGS) were determined by real-time RT-PCR. Relative quantification of gene expression patterns by the ΔΔCt method revealed consistent differences between the six genes (Figure 2C). The levels were normalized against an endogenous housekeeping gene, human large ribosomal protein PO (RPLPO), and results were compared to the Hs294T cell line, given an arbitrary value of 1.

Of the melanoma cell lines studied, the lowest level of expression of the main genes coding for MTA targets was found in HT144. This expression pattern also occurs in the NSCLC cell line Calu-3 where there is also a significantly lower expression of the main MTA target, TS.

Given the patterns of expression obtained, two analyzes are possible. If we restrict the data to those of melanoma cells, it seems possible to distinguish two distinct groups: firstly, the lines (A375, Hs294T and MeWo) with high levels of expression of major drug targets TS, DHFR, and AICART GARFT and on the other hand, those lines (HT144) with low levels of expression of said enzymes.

If we include in this analysis the NSCLC cell line, we can still clearly distinguishing these two groups, but it is possible to classify them based on a single marker: TS expression.

Notably, both groups coincide with the groups identified in the previous restoration pathway assay, based on AICART or GARFT inhibition.

Thus, there appears to be a clear correlation between expression levels of TS inhibition and AICART where: firstly, in cells with low TS, MTA inhibits AICART, while in those cells with high levels TS, MTA does not act on AICART, but on GARFT.

### mTOR Inactivation

**Figure 3:**
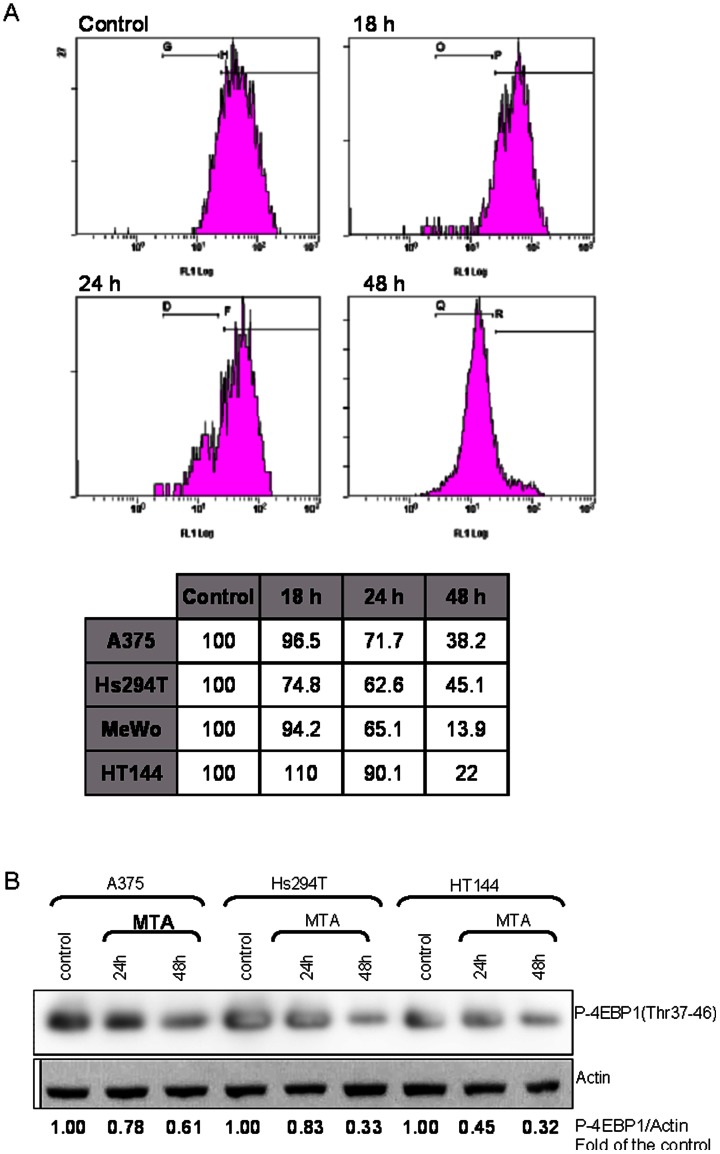
MTA induces a regulation of the PI3K/Akt and TSC2/mTOR pathways. **A)** Analysis by flow cytometry of phosphorylated Akt at Ser473 showed a relevant decrease in protein levels in all cell lines. The histograms shown are an example of the behaviour of the positive population at different MTA-exposure times analysing the A375 cell line. The table below summarizes the results obtained for all the melanoma cell lines. Values are represented as the percentage of the positive population compared with the cell line growing in MTA-free medium which was used as the control. **B)** Immunoblot of the p-4EBP1 protein showing a decrease in this indicator of mTOR activity after MTA exposure. All melanoma cell lines have the same tendency which indicates that MTA has a regulator role in this pathway, allowing the arrest of several survival signalling cascades and processes, prompting cells to die by apoptosis. Values presented are the result of densitometry analysis expressed as the P-4EBP1: actin, the relative content of P-4EBP1 in per unit mass of actin protein and relative to the control.

The blockade of AICART by MTA can be expected to induce an accumulation of ZMP causing the inactivation of the mTOR signalling pathway through AMPK and Akt regulation, because, among other functions, Akt acts as regulator of the mTOR pathway and its inactivation represses the mTOR complex. Despite AICART inhibition not occurring in all cell lines, flow cytometry analysis revealed decreased levels of phosphorylated Akt in all cell lines after MTA treatment (Figure 3A). This MTA-induced hypophosphorylation of Akt might contribute to enhanced apoptotic signalling.

**Figure 4:**
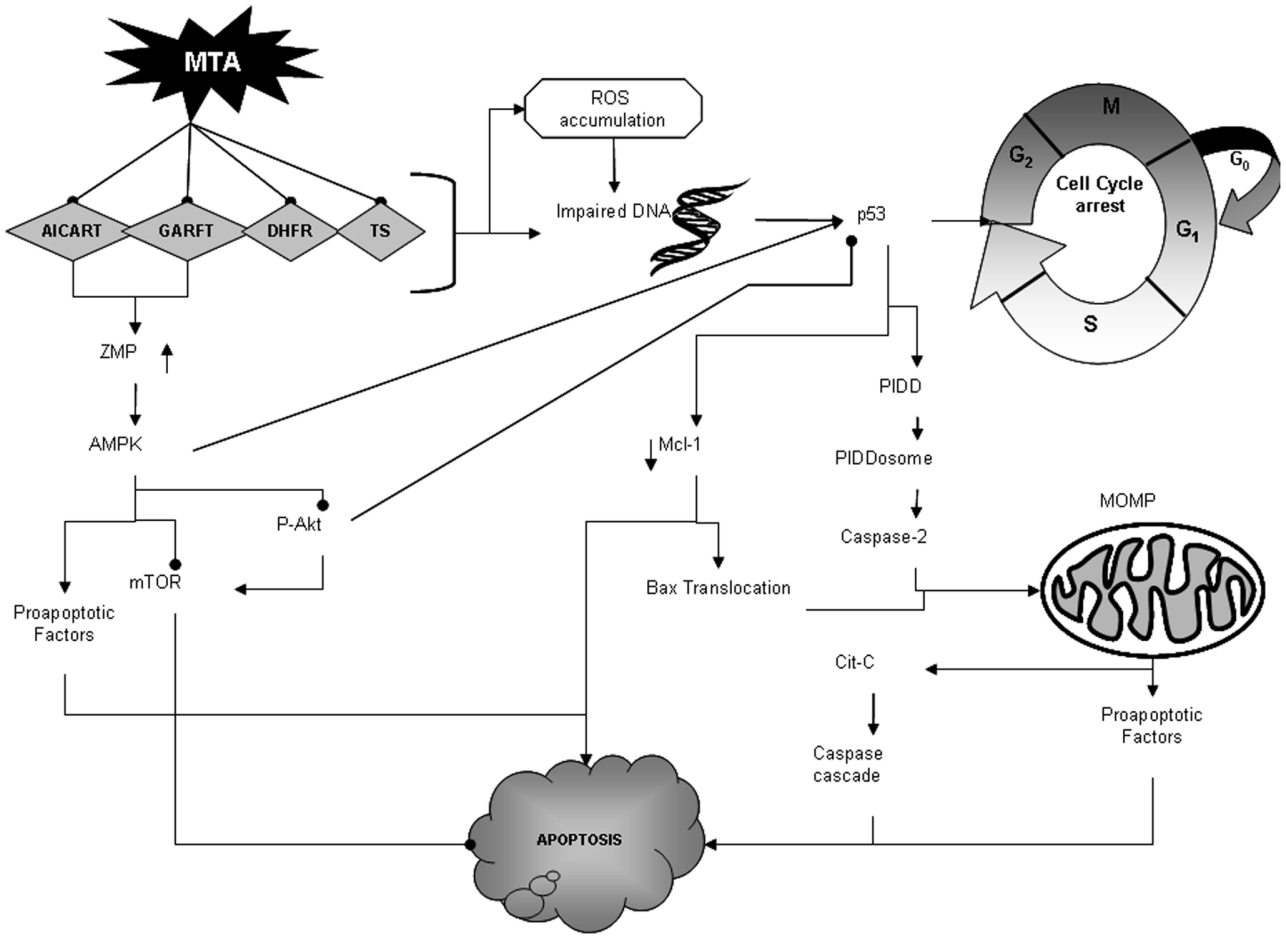
MTA exerts its activity in different pathways increasing the apoptotic stimulus. Firstly, there is an accumulation of the AMP analogue ZMP that induces the activation of the AMPK pathway, starting a cascade of signalling that affects mTOR and PI3P/Akt pathways; mTOR is inactivated and the accumulation of its downstream unphosphorylated substrates facilitates the apoptosis process. Akt also remains inactive, unable to block p53 and to activate mTOR. On the other hand, the inhibition of TS, DHFR, GARFT and AICART induces oxidative stress and DNA damage which in turn is detected by p53 and caspase-dependent and independent mitochondrial apoptosis that is activated as has been previously reported. Together all processes lead to an imbalance between cell death and survival stimuli that result in enhanced apoptotic signalling.

In order to investigate the status of mTOR, we used immunoblotting to determine the expression of one of its substrates, the 4EBP1 protein phosphorylated at threonine 37–46 (p-4EBP1); this is a well known downstream substrate of mTOR which is widely used as indicator of mTOR activity both *in vivo* and *in vitro*. Results showed that after MTA exposure, p-4EBP1 protein levels fell (Figure 3B), pointing to a deregulation of mTOR. Thereafter, 4EBP1 is not recruited by mTOR and would be able to inhibit cell proliferation (Figure 4).

### MTA Resistant Cell Lines

MTA-resistance was generated *in vitro* as described in the Material and Methods section. After 3 months, we obtained MTA-resistant cells able to grow in the presence of 1.7 µM of MTA. Resistance was maintained over several freeze/thaw cycles and was transferred genetically to the subsequent generations. MTA-resistant cell lines were designated as A375-M, Hs294T-M and MeWo-M. On the other hand, we were unable to establish HT144-M (melanoma) or Calu3-M (NSCLC) cell lines; indeed, cells from these lines did not seem to develop resistance to MTA, pointing to a connection between MTA sensitivity, TS expression and/or AICART inhibition (Figure 2C).

Again, we can identify two groups: firstly those cell lines capable of acquiring resistance to the antifolate (A375, Hs294T and MeWo); on the other hand, cell lines that can not (HT144 and Calu-3). And anew, these two groups coincide perfectly with the two groups identified based on the antifolate secondary targets, and based on the expression levels of TS.

Given these results, we can suspect that there is a relationship between the TS expression levels and the ability to acquire resistance to the antifolate: those cells with high TS expression levels are able to develop resistance to the drug after prolonged exposures, while those cells with low TS expression levels are unable to develop resistance to the drug. It is likely that this inability is because in these cells MTA acts on AICART, representing a new point of attack.

### MTA Targets as Genetic Resistance Markers

**Figure 5:**
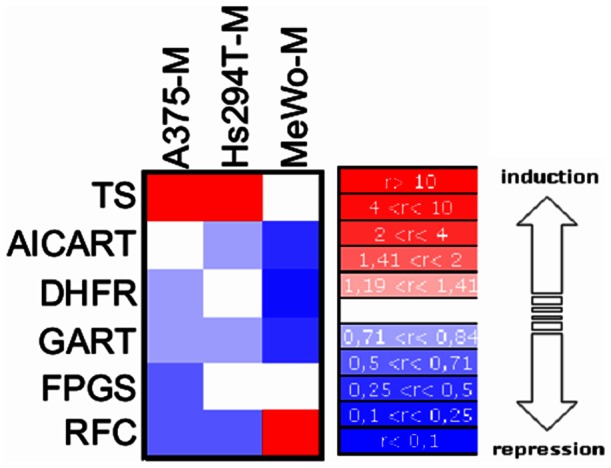
Expression levels of MTA-related enzymes and RFC in MTA-resistant cell lines. Results are represented on a heatmap as the ratio of MTA-resistant vs. -sensitive cell expression. The up-regulation and down-regulation fold changes corresponding to each colour are indicated on the scale on the right of the figure.

Expression levels of MTA-related enzymes and the RFC in MTA-resistant cell lines were assessed by real-time RT-PCR and analysed by the ΔΔCt method, normalizing samples against RPLPO as the endogenous housekeeping gene and comparing results with those for the corresponding sensitive cell lines. TS levels increased in two of the three MTA-resistant cell lines, A375-M and Hs294T-M, while this enzyme seemed not to be particularly relevant in MeWo-M, where the most important changes were a rise in RFC levels and a strong down-regulation of the other three targets of MTA: AICART, DHFR and GARFT (Figure 5). In contrast, FPGS expression was slightly lower in A375-M but no significant changes were detected in Hs294T-M or MeWo-M.

## Discussion

MTA is a relatively new antifolate that is being used in clinical oncology in combination with cisplatin for the treatment of mesothelioma and non-squamous non-small cell lung cancer with encouraging results showing a improved safety profile when administered with vitamin B12 and folic acid [12]. In this context, in recent years, many groups have focused their research on the metabolic pathway affected by MTA. One of the key findings is the effect that this agent has on pathways dependent on tumour suppressor genes whose function is often deregulated during malignant transformation [11].

Results of the present study show that the basic biochemistry of MTA seems to be common to all tumour cells despite small variations that do not affect its cytotoxicity [13]. Concerning the inhibition of the pyrimidine synthesis pathway, we found that MTA targeted primarily TS in all tumour cells, in agreement with previous data on the MTA mechanism. [11], [14], [15] In contrast, regarding the blockade of the purine synthesis pathway there appeared to be some differences between the cell lines and two groups were defined. On the one hand, in the group consisting of HT144 and Calu-3 cells, melanoma and NSCLC cell lines respectively, the addition of AICA and dTh did not suppress and even slightly enhanced the cytotoxicity of MTA; this indicates that MTA interrupt the pathway downstream of the intermediate included in the culture, namely AICART, as previously described in CCRF-CEM, a human lymphoblastic leukaemia cell line [11]. This hypothesis does not, however, rule out an effect on GARFT. On the other hand, in the group formed by A375, Hs294T and MeWo cell lines, the addition of AICA and dTh fully restored the activity of the corresponding pathway suggesting that GARFT, but not AICART, is targeted by MTA.

The importance of which enzyme is the secondary target stems from the fact that AICA is phosphorylated by adenosine kinase to AICA ribotide, also known as ZMP, which mimics the effects of AMP in activating AMPK [11], [13]–[19]. It has been described that the inhibition of AICART by MTA induces an accumulation of ZMP, and thus the activation of the AMPK signalling pathway [11], linked to the important tuberous sclerosis complex 2 (TSC2). TSC2 is able to form a complex with TSC1 and inhibits mTOR activity, which is usually assessed in terms of the phosphorylation status of the downstream substrate 4E-BP1 [11], [20]. This mTOR inhibition leads to negative regulation of several growth and proliferation pathways. At the same time, AMPK is able to inhibit Akt phosphorylation enabling a compensatory pathway that reinforces mTOR inhibition, due to the ability of Akt to phosphorylate and block TSC2 [15], [21]–[23].

Therefore, AICA-mediated AMPK activation enables the inhibition of proliferation and cell cycle progression, via inhibition of both the TSC2/mTOR and PI3K/Akt pathways, and this prompts the up-regulation of p21, p27 and p53, resulting in cell cycle arrest [19], [22].

In this study, differences in terms of the secondary targets of MTA did not seem to have impact on the inhibition of Akt phosphorylation, given that Akt phosphorylation decreased in all cell lines after MTA exposure. A similar observation was made in a previous study exploring the effect of the antifolate Methotrexate (MTX) on various human cancers. On the one hand, MTX was able to inhibit AICART, causing an increase in ZMP levels which potently sensitized tumour cells to apoptosis. At the same time, however, the inhibition of GARFT also induced an AICA riboside-induced ZMP accumulation, suggesting that the effects of GARFT inhibition may induce a more complex cascade related to the purine synthesis pathway [24]. This might explain why, despite the main secondary target of MTA in some tumours seeming not to be AICART but rather GARFT, there could be also an indirect ZMP accumulation leading to Akt dephosphorylation.

Previous research [13] found that in melanoma cells MTA induces a DNA damage leading to p53 up-regulation and cell cycle arrest followed by caspase-dependent and independent apoptosis. Our results indicate that this mechanism is complemented by the activation of an alternative pathway through ZMP-mediated AMPK activation (Figure 4).

We propose that MTA prompted the inhibition of TS, DHFR, GARFT and/or AICART and, consequently, induced oxidative stress and DNA damage leading to the up-regulation of p53 and cell cycle arrest [13]–[15], [22]. At the same time, MTA-inhibited purine biosynthesis by targeting either AICART or GARFT led to an accumulation of ZMP causing the activation of AMPK which, in turn, started a biochemical cascade in which mTOR was inactivated both directly and indirectly by the Akt pathway. These types of regulation together with the DNA-damage increased the expression of checkpoint controls genes such as p53 causing the cell cycle arrest [13], [21]. On the other hand, the AKT/PI3K pathway plays a central role in linking various cellular stimuli to a broad range of cellular functions and its inactivation could result in stronger apoptosis stimuli through caspase-dependent and independent factors [19], [21], [22]. The overall signalling produces sustained cell proliferation arrest, which can ultimately lead to a loss of viability due to the triggering of apoptotic pathways.

In order to correlate differences in MTA secondary targets with the expression of related enzymes, basal mRNA expression levels of MTA-related enzymes (TS, DHFR, AICART, GARFT, FPGS and RFC) were assessed by one-step real-time RT-PCR. Interestingly, cell lines whose secondary target was AICART (HT144 and Calu-3) had significantly lower TS expression. TS expression has been widely studied as a prognostic factor in MTA-treated tumours and has previously been related to the sensitivity of tumour cells to antifolates [15]. Similarly, studies on pancreatic cancer cells have demonstrated that low levels of TS, DHFR and GARFT make cells more sensitive to MTA treatment, whereas a lower sensitivity could be explained by the higher expression of the MTA targets [14]. In this case, although the low target expression not seeming to compromise the effect of MTA, it appeared to be a hallmark of those cells with AICART as secondary target.

Likewise, this TS low expression seems to be a characteristic feature of those cell lines capable of acquiring drug resistance after exposure. As a result of our attempts to develop MTA-resistant cell lines, we obtained firstly, three well established resistant cell lines that were named: A375-M, Hs294T-M and MeWo-M; and secondly, a failure to develop resistance in HT144 or Calu-3 cell lines with the employed method. Assuming that the intrinsic genetic characteristics of the cells causing the tumour are what define the innate ability of the tumour to resist treatment, drug resistance has a significant hereditary component. Consistent with the aforementioned scenario, the intrinsic characteristics of the lines that are capable of developing resistance to MTA appeared to differ from those lines unable to develop such resistance, in terms of whether low basal expression of enzymes related to MTA was correlated with inhibition of AICART. This correlation was observed in both HT144 and Calu-3 and, moreover, these were the lines that were the most sensitive to MTA, in the sense that they were unable to develop drug resistance. It is therefore feasible that the inherent characteristics of each cell type have a significant role and, in this case, the element acted on is AICART. This is a positive factor, the action on AICART strengthening the effect of the antifolate by bringing a new point at which to block the synthesis of nucleotides and strength of activation of the AMPK route.

Current treatment regimens are cyclical, and are based on allowing the patient's recovery from the effects caused by such aggressive treatment. However, these schemes also enable the adaptation of the cells to the agent to which the tumour is being treated thus favouring a resistance due to prolonged exposure to the drug. The genetic changes that tumour cells acquire during treatment serve to nurture and help develop resistance that is specific to each drug. Some genetic changes attributable to drug exposure were found in these new resistant strains (A375-M, Hs294T-M, and MeWo-M). Specifically, the acquired changes were fundamentally an increased expression of TS, the main target of MTA, in A375-M and Hs294T-M, and a decreased expression of secondary targets in the case of MeWo-M. TS expression has previously been associated with sensitivity to MTA and is one of the main determinants of acquired resistance to antifolates in other tumour types such as lung cancer. In addition, patients with this disease that show low levels of TS expression have a better prognosis [16]–[18].

It should be kept into account that the expression of DHFR, TS and GARFT are correlated with each other; furthermore, there is a link between TS and p53 status, as described in colon cancer. It has been found that patients with p53-wt have lower levels of TS expression and therefore a better prognosis; on the contrary, patients whose p53 is mutated (p53-mt) exhibit elevated levels of TS [18]. MeWo is p53-mt, and this could explain why MeWo provided the highest inherent levels of TS and one could hypothesise that this innate TS high expression is the reason why the acquisition of resistance to MTA is developed by a mechanism that differed significantly from that seen in A375 and Hs294T where there was a clear increase in TS. Further studies are needed in a large cohort of patients treated with MTA to state that an increase in the expression levels of TS is an optimal marker for identifying tumours that have been made resistant to treatment with this antifolate. Although *in vitro* studies suggest it.

We can conclude that MTA exerts its effects on a wide spectrum of tumours, independent of the presence of mutations in BRAF^V600E^, LKB1, p53, CDKN2A, PTEN and NF1 (Table 1). That MTA seems to be able to simultaneously regulate multiple pathways related to the progression of melanoma, making it a suitable treatment option for patients with metastatic melanoma. And finally, that tumour cell sensitivity to MTA appears to follow a genetic pattern where high TS expression correlates with a lack of AICART inhibition, hindering the development of resistance to MTA. This correlation between intrinsic genetic characteristics of tumours and the inhibition of AICART or GARFT by MTA could be the basis of a genetic approach to determining the sensitivity of tumour cells to MTA and their ability to become resistant to this antifolate.
